# Mining oomycete proteomes for phosphatome leads to the identification of specific expanded phosphatases in oomycetes

**DOI:** 10.1111/mpp.13425

**Published:** 2024-03-10

**Authors:** Min Qiu, Yaru Sun, Siqun Tu, Huaibo Li, Xin Yang, Haiyang Zhao, Maozhu Yin, Yaning Li, Wenwu Ye, Ming Wang, Yuanchao Wang

**Affiliations:** ^1^ Department of Plant Pathology Nanjing Agricultural University Nanjing Jiangsu China; ^2^ The Key Laboratory of Plant Immunity Nanjing Agricultural University Nanjing Jiangsu China; ^3^ Key Laboratory of Soybean Disease and Pest Control (Ministry of Agriculture and Rural Affairs) Nanjing Agricultural University Nanjing Jiangsu China

**Keywords:** metallo‐dependent protein, phosphatase, oomycete, phosphatase, *Phytophthora sojae*

## Abstract

Phosphatases are important regulators of protein phosphorylation and various cellular processes, and they serve as counterparts to kinases. In this study, our comprehensive analysis of oomycete complete proteomes unveiled the presence of approximately 3833 phosphatases, with most species estimated to have between 100 and 300 putative phosphatases. Further investigation of these phosphatases revealed a significant increase in protein serine/threonine phosphatases (PSP) within oomycetes. In particular, we extensively studied the metallo‐dependent protein phosphatase (PPM) within the PSP family in the model oomycete *Phytophthora sojae*. Our results showed notable differences in the expression patterns of PPMs throughout 10 life stages of *P. sojae*, indicating their vital roles in various stages of oomycete pathogens. Moreover, we identified 29 PPMs in *P. sojae*, and eight of them possessed accessory domains in addition to phosphate domains. We investigated the biological function of one PPM protein with an extra PH domain (PPM1); this protein exhibited high expression levels in both asexual developmental and infectious stages. Our analysis confirmed that PPM1 is indeed an active protein phosphatase, and its accessory domain does not affect its phosphatase activity. To delve further into its function, we generated knockout mutants of PPM1 and validated its essential roles in mycelial growth, sporangia and oospore production, as well as infectious stages. To the best of our knowledge, this study provides the first comprehensive inventory of phosphatases in oomycetes and identifies an important phosphatase within the expanded serine/threonine phosphatase group in oomycetes.

## Graphical abstract

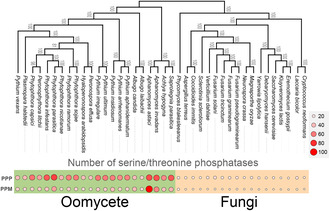

## 1 INTRODUCTION

Phosphorylation and dephosphorylation processes play a crucial role in maintaining the molecular and cellular signalling homeostasis. Protein kinases and phosphatases facilitate the transfer of phosphate between substrates. Phosphorylation regulates over 70% of cellular proteins, as revealed by human genome analyses, which identified 518 kinases that are either tyrosine (Tyr) kinases or serine/threonine (Ser/Thr) kinases. In contrast, the total number of Tyr phosphatases and Ser/Thr phosphatases is approximately 137, significantly lower than the aforementioned kinases (Ardito et al., 2017; Manning et al., 2002). Consequently, protein phosphatases must be tightly regulated, particularly in terms of their balance with kinase counterparts, depending on the specific substrate involved in a wide range of cellular processes. A single phosphatase can modify multiple substrate proteins, while a single substrate protein can be regulated by several phosphatases (Jin & Pawson, 2012).

Protein phosphatases play a crucial role in catalysing the hydrolysis of phosphate groups from proteins, thus reversing the actions of protein kinases. Together, protein kinases and protein phosphatases act as key regulators of all aspects of eukaryotic cell physiology (Brautigan & Shenolikar, 2018). While protein kinases primarily consist of a single protein structural fold in their catalytic domains (Hanks & Hunter, 1995), protein phosphatases have diverse origins with distinct sequence and structural characteristics, despite sharing the same functions (Bheri et al., 2021). Additionally, some of the protein folds exhibit other hydrolase activities or are present in non‐enzymatic proteins (Chen et al., 2017). The nomenclature of phosphatases follows different rules to kinases. For instance, the protein tyrosine phosphatases (PTP) superfamily, including tyrosine‐specific (classical PTP), dual specificity (DSP), low molecular weight PTP (LMWPTP) and CDC25 phosphatases, is named based on characteristic motifs identified in phosphate protein sequences, such as the highly conserved active site motif Cys‐X_5_‐Arg (CX_5_R) (Maehama et al., 2001). On the other hand, alkaline phosphatases (AP) are named after their hydrolysis activities at basic pH values (Sharma et al., 2014).

Protein phosphorylation can occur on nine different amino acids: tyrosine, serine, threonine, cysteine, arginine, lysine, aspartate, glutamate and histidine. Among these, serine, threonine and tyrosine phosphorylation are the most prevalent in eukaryotic cells, with serine being the primary target (Hunter, 2022). In humans, the distribution of phosphorylation on serine, threonine and tyrosine is approximately 86.4%, 11.8% and 1.8%, respectively (Olsen et al., 2006). Dephosphorylation enzymes for these three amino acids can be grouped into four categories based on their unique catalytic signatures, domain sequences and substrate preferences. The PPP (phosphoprotein phosphatase) and PPM (metallo‐dependent protein phosphatase) families are responsible for the majority of dephosphorylation of serine and threonine. It is intriguing to note that, despite having highly similar structures at their catalytic centres, the PPM and PPP families have evolved from two separate ancestral genes with distinct sequences (Das et al., 1996). On the other hand, the PTP (protein tyrosine phosphatase) family, characterized by the catalytic signature CX_5_R, demonstrates substantial diversity in domain structure and substrate preference. Classical PTPs target phospho‐Tyr, while dual specificity phosphatases (DSPs) target both phospho‐Tyr and phospho Ser/Thr. Additionally, some members of the PTP family have been found to dephosphorylate complex carbohydrates, mRNA and phosphoinositides (Alonso et al., 2016). Finally, the recently classified aspartate‐based phosphatases, including FCP (TFIIF‐associating component of RNA polymerase II CTD phosphatase)/SCP (small CTD phosphatase) and haloacid dehalogenase (HAD) phosphatases, rely on an aspartic acid signature (DXDXT/V) for catalysis (Schweighofer & Meskiene, 2015). During dephosphorylation reactions, PTPs and DSPs use an active cysteine residue, while FCP/SCP and HAD phosphatases employ an aspartate‐based mechanism. PPP and PPM enzymes require metal ions to carry out their catalytic activity (Brautigan, 2013).

Oomycetes, currently classified as Stramenopiles, are eukaryotic microbes that physically resemble filamentous fungi; however, they are phylogenetically distinct and possess unique biological, genetic and physiological features. Oomycetes are filamentous microorganisms found ubiquitously, including several important pathogens that infect a wide range of animals and plants. The life stages of oomycetes include vegetative hyphae, sexual structures and asexual spores, including flagellated zoospores (Judelson & Blanco, 2005). Previous studies have demonstrated the involvement of phosphatases in the vegetative growth and sporangial development of oomycetes (Ah Fong & Judelson, 2003; Qiu et al., 2021; Zhao et al., 2011). While a comprehensive investigation of kinases has been conducted in *Phytophthora infestans*, examining oomycete‐specific innovations (Judelson & Ah‐Fong, 2010), there is a lack of thorough and systematic research on phosphatases in oomycetes. In this study, we present a comprehensive catalogue and classification of oomycete phosphatases, focusing on the phosphatome of one oomycete, *Phytophthora sojae*, which is a pathogen causing soybean root rot. We specifically highlight oomycete‐specific features, the expression pattern of phosphatases, the presence of novel domains and accessory domains and the biological role of a Ser/Thr phosphatase.

## 2 RESULTS

### 2.1 Definition of the phosphatomes in oomycete species

The phosphatomes in oomycete species are defined as the set of phosphatases (protein or nonprotein substrates) that contain known phosphatase catalytic domains. Although the naming rules for known phosphatases across Eukaryota may vary in protein names, their catalytic domains remain conserved. Therefore, these shared catalytic domains were compiled from shared phosphatase families, including those found in plants, fungi and mammals (Figure S1). The catalytic domains responsible for serine, threonine or tyrosine phosphorylation include PF00102‐PTP, PF00581‐CDC25, PF00782‐DSP, PF01451‐LMWPTP, PF04722‐SSU72, PF00149‐PPP, PF00481/PF07228‐PPM, PF04181‐RTR1, PF03031/PF13344‐HAD. The remaining folds either have nonprotein substrates, primarily lipids (PF06602‐Myotubularin, PF02383‐Sac, PF14566‐Paladin) or monoesters (PF00245‐AP) or have no known substrate (PF00300/PF00328‐HP) (Figure S1).

To gain a comprehensive understanding of the phosphatase family in oomycetes, the conserved catalytic domains shown in Figure S1 were used to identify all members of the phosphatase family from a total of 22 oomycete species. This selection comprises six *Phytophthora* (*Phytophthora ramorum*, *Phytophthora sojae*, *Phytophthora capsici*, *Phytophthora nicotianae*, *Phytophthora parasitica* and *Phytophthora infestans*), one *Peronophythora* (*Peronophythora litchii*), one *Hyaloperonospora* (*Hyaloperonospora arabidopsidis*), one *Peronospora* (*Peronospora effusa*), one *Plasmopara* (*Plasmopara halstedii*), six *Pythium* (*Pythium vexans*, *Pythium aphanidermatum*, *Pythium arrhenomanes*, *Pythium insidiosum*, *Pythium irregulare* and *Pythium ultimum*), two *Albugo* (*Albugo candida* and *Albugo laibachii*), one *Saprolegnia* (*Saprolegnia parastica*), one *Achlya* (*Achlya hypogyna*) and two *Aphanomyces* species (*Aphanomyces astaci* and *Aphanomyces invadans*). In addition, 18 fungal species covering a broad taxonomic range were also included in the analysis.

**FIGURE 1:**
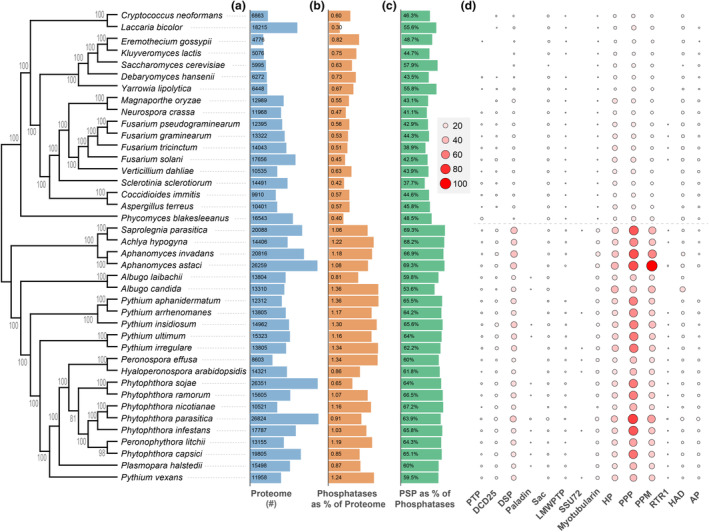
Numbers of phosphatases in oomycetes and fungi. Phylogenetic analysis of 22 oomycetes and 18 fungi, a maximum‐likelihood phylogeny was generated based on single‐copy genes obtained from OrthoFinder. (a) Proteomes sizes of the 22 oomycetes and 18 fungal species. (b) Ratios of phosphatases to total proteome in each species. (c) Ratios of protein Ser/Thr phosphatases (PSP) to total phosphatases in each species. (d) Classification of phosphatases by types of catalytic domains. The size and colour of the circles are determined by the number of genes. Larger, darker red circles represent a greater number of genes.

Using the conserved catalytic domains as a guide, 4861 phosphatase genes were identified from 22 oomycete and 18 fungal species (Table S1). This revealed that oomycetes have a higher number of phosphatases compared to fungi. On average, oomycete species contained 174 phosphatases, whereas fungal species had an average of 57 phosphatases (Table S1). The number of phosphatases within different oomycete species ranged from 100 to 300, while in fungal species, it was approximately 30 to 80 (Table S1). To ascertain whether the increased number of phosphatases in oomycetes was due to larger proteomes, we calculated the relative ratios of phosphatases to total proteomes for each species. Interestingly, we found that oomycetes consistently exhibited higher ratios of phosphatases, ranging from 0.65% to 1.36%, with an average ratio of 1.1%. In contrast, fungal species displayed lower percentages of phosphatases, ranging from 0.3% to 0.8%, with an average ratio of 0.62% (Figure 1a–c; Table S1). These findings suggest that oomycetes possess a more extensive repertoire of phosphatases compared to fungal species, which may reflect differences in the biology and evolution of these two groups of pathogens.

### 2.2 Oomycetes have expanded numbers of serine/threonine protein phosphatases

To further investigate the increased number of phosphatases in oomycetes, we then conducted an analysis of the phosphatase subgroups within all included species. All the phosphatases were grouped into 14 families based on their catalytic domains (Figures 1d and S1) and summarized in Table S2.

Based on the distribution of phosphatases in oomycetes, the DSP, PPP and PPM were the three subfamilies with the highest number of phosphatases in oomycetes, accounting for approximately 60% of all phosphatases (Figure 1c). Compared with fungi, all oomycetes displayed a significant increase in the number of phosphatases in these three subfamilies (Figure 1c), suggesting that the expansion of these subfamilies was probably an early event in oomycete evolution. As PPP and PPM are Ser/Thr‐specific phosphatases while DSP have activities in either Ser/Thr or Tyr (Mumby & Walter, 1993; Uhrig et al., 2013), it can be concluded that oomycete phosphatomes may display expansion mainly on protein Ser/Thr phosphatases (PSP).

So far, most living organisms have a proportionately larger number of kinases than phosphatases (Bhaskara et al., 2019; Chen et al., 2017). To match this abundance of kinases, it is highly possible that one phosphatase is involved in the dephosphorylation of two or more substrates. Therefore, the specificity and precise regulation of phosphatases is an important process to allow for the dynamic control of protein phosphorylation. The PSPs use different ways to regulate their enzymatic activities. For the PPP family, they usually associate with a great variety of regulatory subunits and work in multimeric proteins. In contrast to the PPP family, PPMs are monomeric enzymes and it will be more straightforward to analyse the PPM family for expanded events.

**FIGURE 2:**
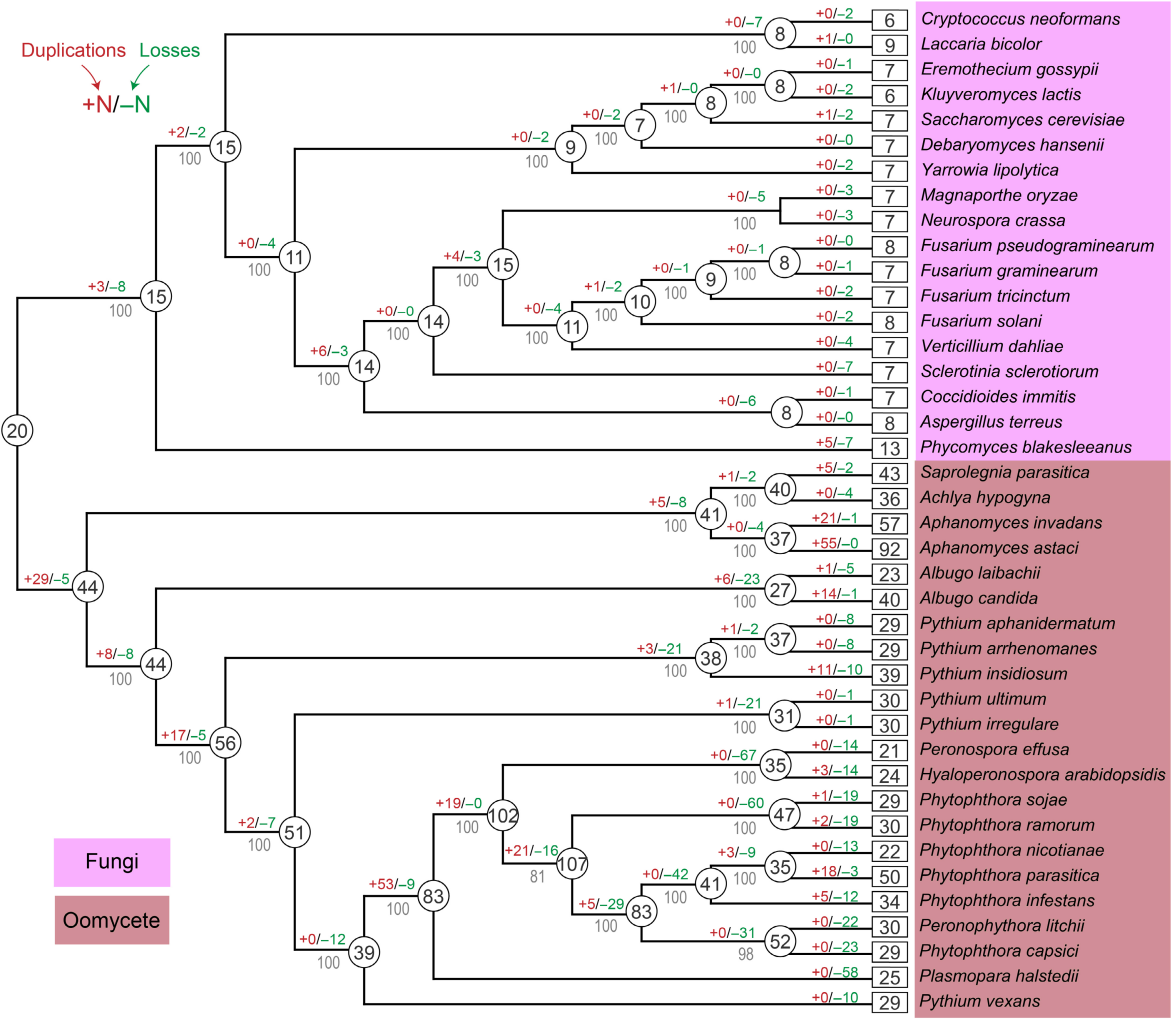
Phylogenetic tree correspondence with gene duplications and losses of the metallo‐dependent protein phosphatase (PPMs) in oomycete and fungal species. We tallied the count of PPM genes acquired and discarded for each orthologous group within the 22 oomycete and 18 fungal species of the phylogenetic tree. The number on the initial branch represents the quantity of genes present in the most recent common ancestor. Numbers with a plus sign in front signify the number of genes duplicated (marked with red), while those with a minus sign indicate the number of genes lost (marked with green).

To systematically study the expansion events in oomycete and fungal species, we used the PPM protein sequences obtained from 22 oomycete and 18 fungal species (Table S2) to analyse the gene duplications and losses within the PPM family. The accuracy of gene family evolutionary analysis relies heavily on having a correct species phylogeny. Moreover, individual gene trees may not necessarily represent the true relationships between species. To establish a reliable species phylogeny, we identified single‐copy genes from the 22 oomycete and 18 fungal species using OrthoFinder (Emms & Kelly, 2019). Using these single‐copy genes, we constructed the species phylogeny through a maximum‐likelihood approach, and we assessed its robustness with 1000 bootstrap replicates. The obtained species phylogeny received strong support, with bootstrap values exceeding 80% for all nodes. This phylogeny closely resembles the known tree of life topology, clearly distinguishing oomycetes from fungi (Figure 2). By projecting gene duplications and losses onto the species phylogeny, we gained a comprehensive and dynamic understanding of PPM evolution. We observed that a high number of duplications occurred in the common ancestor of oomycetes, as well as in the genera of *Peronospora* and *Phytophthora* species. However, within the oomycete species having expansion events, an equally high number of losses balances the high number of duplications. As a result, most oomycete species possess a similar number of PPMs (Figure 2).

### 2.3 Conserved core sites in catalytic domains of metallo‐dependent protein phosphatases

**FIGURE 3:**
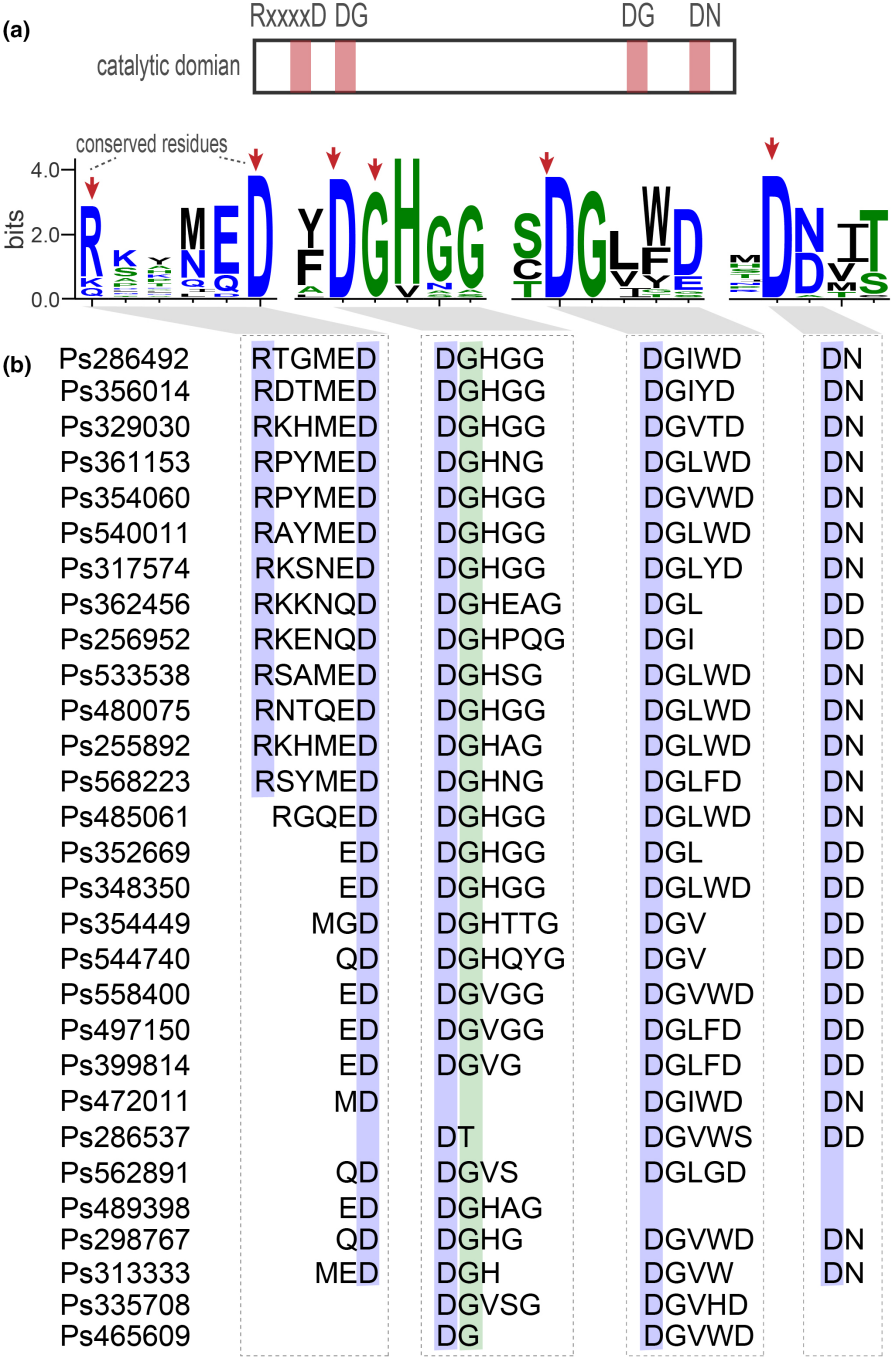
Analysis of conserved catalytic core sites in *Phytophthora sojae* metallo‐dependent protein phosphatase (PPMs). (a) The characteristic sequence motifs within the catalytic domains of 29 PPMs from *P. sojae*. The height of symbols within the stack indicates the relative level of conservation of each amino acid at that position. R, arginine; x, any amino acid; D, aspartic acid; G, glycine; H, histidine; L, leucine; W, tryptophan; N, asparagine. Red arrows, residues that contribute to metal coordination and phosphate binding. (b) The conserved residues identified in each PPM. Residues that contribute to metal coordination and phosphate binding are marked with blue or green.

It has been widely demonstrated that PPMs require binding of Mg^2+^ or Mn^2+^ in their active centre for activity. PPMs share nearly identical core domains from prokaryotic to eukaryotic species, with the active site residues highly conserved (Shi, 2009). To explore whether the expanded PPM groups in oomycetes still contain conserved catalytic core sites, we analysed the significant features of the alignment catalytic domains in *P. sojae*, one of the model species for oomycete research. We observed four characteristic sequence motifs within the conserved catalytic domain: RxxxxD, DGHGG, DGLWD and DN/D (Figure 3a). These four motifs contain all the reported conserved residues: the R and D from the RxxxxD motif; the D and G from the DGHGG motif; the D from the DGLWD motif; the D from the DN/D motif (Figure 3a, indicated by red arrows).

To further specify the conserved residues in each *P. sojae* PPM, we searched for these conserved residues in the aligned sequences. We found that the majority of proteins contain all the conserved residues, although a few may have deletions or mutations in the core sites. Of the six core residues, residue D (aspartic acid) was the most abundant and also appeared to be the most conserved. Of the 29 PPMs, 23 PPMs were conserved in all four D residues. The residue G (glycine) had similar conservation and appeared in 27 PPMs. The residue R (arginine) had the least conservation and appeared in only 14 PPMs (Figure 3b). The differences in amino acid conservation might be associated with differences in substrate specificity or substrate binding among PPMs. PPMs with high conservation of D and G might share common substrates or functions, while those with less conservation of these residues could have unique roles.

### 2.4 Clustered transcriptional patterns of metallo‐dependent protein phosphatases

**FIGURE 4:**
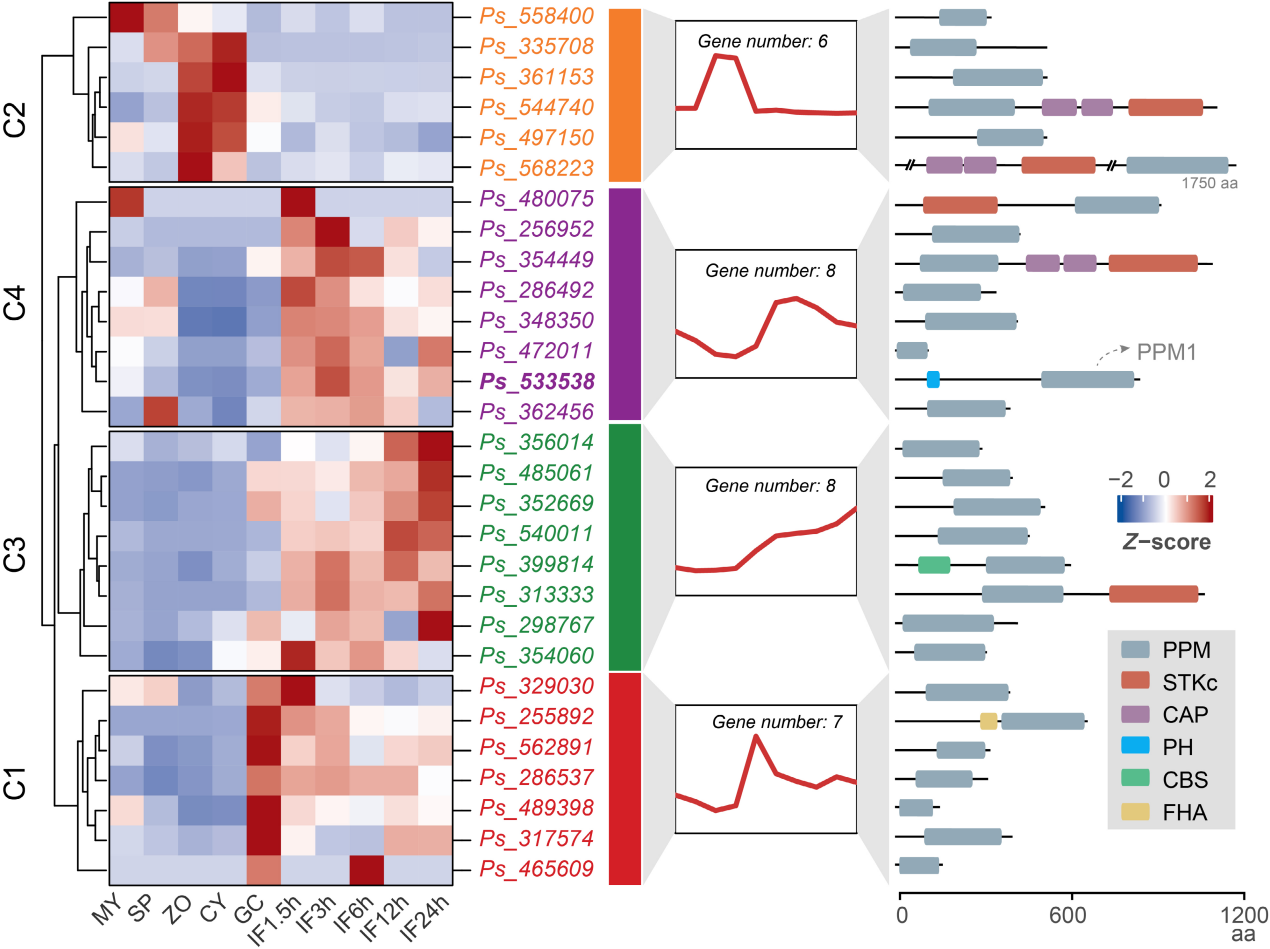
Hierarchical clustering of *Phytophthora sojae* metallo‐dependent protein phosphatase (PPMs) in transcriptional patterns. Hierarchical clustering of transcription levels in 10 life stages of the genes from *P. sojae* PPM family. The 10 life stages of *P. sojae* examined were five asexual developmental stages mycelia (MY), sporangia (SP), zoospore (ZO), cyst (CY), and germinating cyst (GC), and five infectious stages at 1.5, 3, 6, 12 and 24 h after *P. sojae* inoculation on soybean leaves (IF1.5 h, IF3h, IF6h, IF12h and IF24h as abbreviations). The right panel of this figure shows the domain structures of each protein in *P. sojae* PPM family. The proteins from PPM subgroup contain two types of PPM domains (PF00481 and PF07228), that belong to the protein phosphatase 2C (PP2C) family (Bradshaw et al., 2017), thus we unified them as PPM domain in this study.

To determine how PPMs are expressed, we analysed their expression patterns in transcriptome databases from *P. sojae*. We examined 10 life stages: five development stages and five infection stages (Ye et al., 2011). Figure 4 illustrates the expression patterns of genes in the PPM family, which have been ordered based on hierarchical clustering. Red and blue colours indicate upregulation and downregulation, respectively, compared with the mean expression levels across the 10 stages. The middle portion of the figure displays the average expression patterns within each clustered group. Based on hierarchical clustering, the 29 PPMs from *P. sojae* can be clustered into four groups, C1 to C4. The first group (C1) comprises seven genes that exhibit high expression in the germinated cyst stage but decrease gradually during the infection stages. The second group (C2) consists of six genes that are most highly expressed during the zoospore and cyst stages. The third group (C3) includes eight genes that are highly expressed during the infection stages. The fourth group (C4) contains eight genes that exhibit moderate expression during the mycelia and sporangia stages, followed by a decrease in the zoospore, cyst and germinated stages, and then they are highly expressed during all infection stages (Figure 4).

These clustered expression groups indicate that the PPM family exhibits notable differences throughout these 10 life stages, suggesting important roles in various stages of *P. sojae*. Of these groups, the genes from group C4 are of particular interest due to their high expression levels in most stages. They exhibit moderate expression levels in mycelia or sporangia, followed by a decrease in expression during the zoospore, cyst and germinated cyst stages and a subsequent increase in expression during the infection stages. This type of extensive expression pattern indicates that these genes have roles in multiple life stages of *P. sojae*.

### 2.5 Accessory domains in metallo‐dependent protein phosphatases

In some cases, when enzymes within the same family display remarkably similar mRNA abundance levels across distinct stages, a concern arises of how these enzymes achieve their specificity. Phosphatases employ diverse strategies to ensure enzymatic specificity, including considerations of localization, regulatory proteins, accessory domains and other factors (Brautigan, 2013). Therefore, in light of the previous analysis of clustered expression groups within the PPM family, identifying a potentially significant PPM gene necessitates a consideration of the specificity of proteins within a gene family that share similar gene expression patterns. In the case of the PPM family, they contain additional domains that may play a role in determining substrate specificity (Moorhead et al., 2009). Next, we analysed the full protein sequences of *P. sojae* PPMs for functional domains and found that 8 out of 29 PPMs contain accessory domains in addition to the phosphatase domains (Figure 4). These domains include STKc (Catalytic domain of Serine/Threonine Kinase), CAP (Catabolite Activator Protein, also known as cAMP receptor protein), PH (Pleckstrin Homology), CBS (Cystathionine Beta Synthase) and FHA (Fork Head Associated). The detailed information is summarized in Table S3.

The STKc domains are classic kinase domains that are involved in phosphorylation processes. It is intriguing that these STKc‐containing PPMs, Ps354449, Ps544740, Ps568223, Ps313333 and Ps480075 (Figures 4 and S2), are capable of both phosphorylation and dephosphorylation within a single protein, indicating their concurrent kinase and phosphatase activities. The accessory CAP domain, which is always present in the PPM family along with the STKc domain (Figure 4), is predicted to have cyclic nucleotide‐binding activity, suggesting that these STKc domains may be cAMP‐ and cGMP‐dependent protein kinases. Furthermore, these STKc‐containing PPMs were assigned into different groups based on their expression patterns.

Ps544740 and Ps568223 were clustered into the second group (C2) and had high mRNA levels during zoospore and cyst stages. Ps480075 and Ps354449 both had high mRNA levels during early infection stages and Ps480075 also show high mRNA levels during the mycelial stage. Ps313333 was in the third group (C3) and had high mRNA levels across infection stages. Their expression patterns were distributed across all life stages with few overlaps, implying the existence of precise mechanisms regulating these bifunctional enzymes.

The remaining three accessory domains, PH, CBS and FHA, were identified only once in these PPMs (Figure 4). The PH domain is reported to bind phosphoinositides, subunits of heterotrimeric G proteins and protein kinase C. Through these interactions, PH domains play a role in recruiting proteins to different membranes, thus targeting them to appropriate cellular compartments or enabling them to interact with other components of the signal transduction pathways (Powis et al., 2023). Therefore, the accessory PH domain may help its associated enzymes locate in appropriate membranes. The CBS domain is reported to bind ligands with an adenosyl group, such as AMP and ATP. Upon binding to these different ligands, the CBS domains regulate the activity of their associated enzymatic domains (Janosik et al., 2001; Scott et al., 2004). This suggests that CBS domains may play a regulatory role, making proteins sensitive to adenosyl‐carrying ligands. The FHA domain displays specificity for phosphothreonine‐containing epitopes but can also recognize phosphotyrosine with relatively high affinity (Wang, 2023). Regarding the PPMs, which are Ser/Thr phosphatases, the FHA domain may be specific to the dephosphorylation of threonine.

### 2.6 PPM1 was an active phosphatase with enzyme activity

Based on the data obtained above, of the eight PPMs with accessory domains, five PPMs (Ps354449, Ps544740, Ps568223, Ps313333 and Ps480075) were bifunctional enzymes with concurrent kinase and phosphatase activities. For the remaining three PPMs that possessed accessory domains (PH, CBS and FHA), existing literature indicates that the PH domain can bind to phosphoinositides (Powis et al., 2023), the CBS domain has an affinity for ligands containing an adenosyl group, such as AMP and ATP (Janosik et al., 2001, Scott et al., 2004), and the FHA domain exhibits specificity for phosphothreonine‐containing epitopes while also having a relatively high affinity for phosphotyrosine (Wang, 2023). Therefore, it is highly plausible that these accessory domains (PH, CBS and FHA) within PPM genes may play a crucial role in determining substrate specificity, probably due to their involvement in ligand recognition. These data indicated the precise regulating mechanisms underlying these seven PPMs and suggested their importance in life activities. Among these PPMs, the fourth group (C4), which we were most concerned about due to its extensive expression patterns, contained only one gene, Ps533538 (referred to as PPM1 hereafter), with an additional N‐terminal PH domain, and this additional domain existed only once in the *P. sojae* PPM family (Figure 4). Therefore, PPM1 was subjected to further investigation of its biological roles.

**FIGURE 5:**
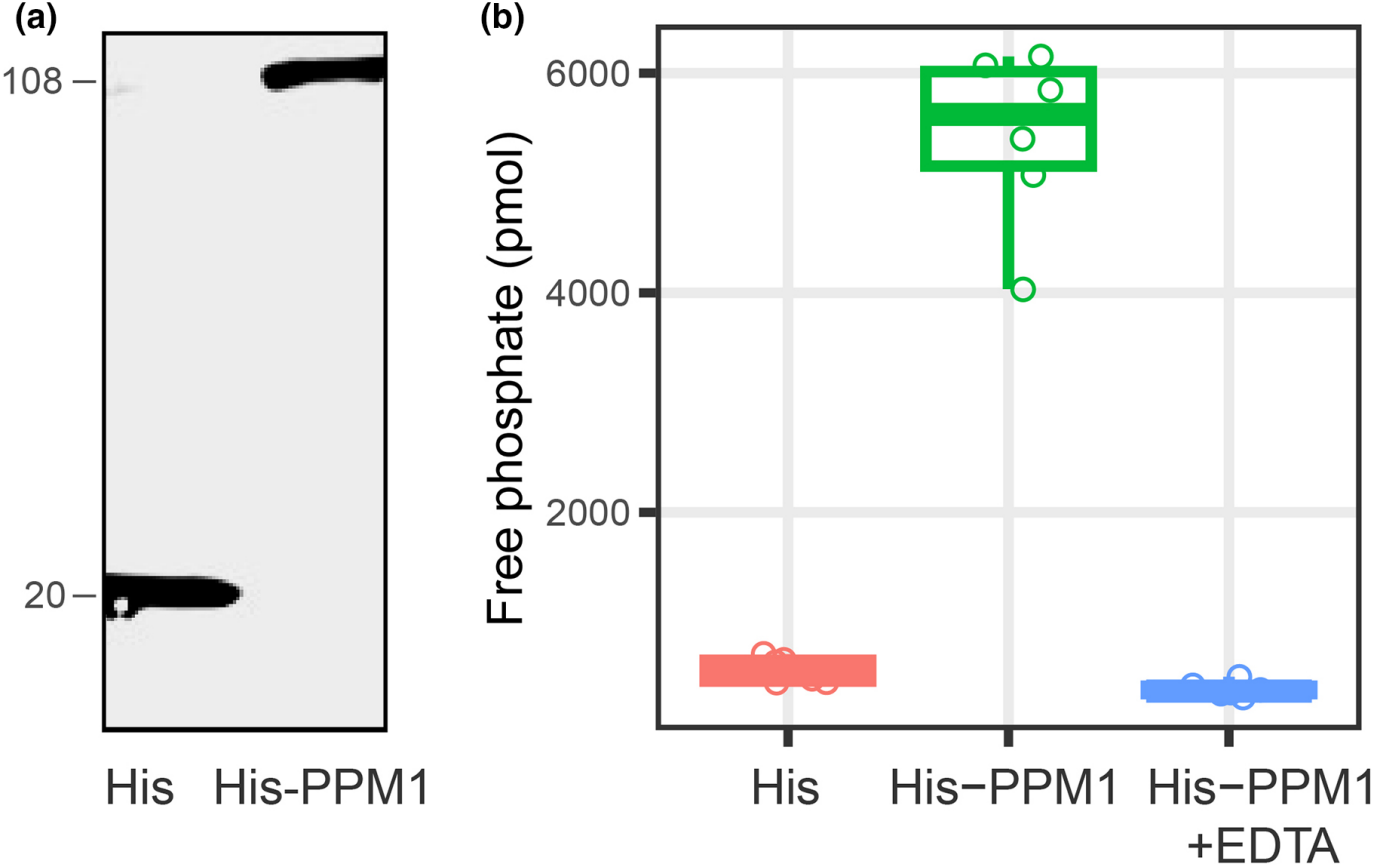
Phosphatase activity of *Phytophthora sojae* PPM1. (a) The purified protein of negative control (His) and His tag‐fused PPM1 (His‐PPM1). The empty pET32a vector contains Trx tag, S tag and His tag, thus the negative control (His) encodes a 20 kDa protein. The full length of *PPM1* was 2418 bp and the His‐PPM1 vector encodes a fusion protein about 108 kDa. The presence of His‐tagged proteins was detected by western blot analysis using an anti‐His antibody. (b) The amount of free phosphate that released by negative control (His), His tag‐fused PPM1 (His‐PPM1) and His‐PPM1 mixed with chelating agent (EDTA). The experiments were repeated three times with similar results (Table S5).

To determine whether the accessory domain has an effect on its phosphate activity, we purified the full protein of PPM1 (His‐PPM1) and the PPM1 protein without its PH domain (His‐dePH) (Figures 5a and S3a), and an absorbance‐based detection of phosphatase activity was used to measure its phosphatase activity. The activity of His‐PPM1 or His‐dePH were measured by the abundance of the free phosphatase. As shown in Figures 5b and S3b, the full protein of PPM1 (His‐PPM1) and the PPM1 protein without its PH domain (His‐dePH) both showed high free phosphatase activity, while the mock (His) had very low release of free phosphate. Because the phosphatase activity of PPMs is dependent on the binding of Mg^2+^ or Mn^2+^ ions in their active centre, EDTA can efficiently inhibit the activity of PPM families by chelating positively charged ions. After adding EDTA to His‐PPM1 or His‐dePH protein, the amount of free phosphate decreased to a similar level as the mock (Figures 5b and S3b), further verifying that the release of free phosphate was dependent on the activation of PPM. This data confirms that PPM1 is an active protein phosphatase and its accessory domain has no impact on its phosphatase activity. Moreover, the activity is highly dependent on its cation‐binding activity.

### 2.7 Deletion of PPM1 significantly affected mycelial growth, sporangia and oospore production and virulence of *P. sojae*

For further investigation of the biological role of PPM1 in *P. sojae*, knockout mutants were generated by CRISPR/Cas9 mediated technology, and the phenotypes of three knockout mutants, *PPM1*‐KO1, *PPM1*‐KO2 and *PPM1*‐KO3, were analysed (Figure S4). The recipient wild‐type strain (WT) and empty vector lines (EV in which the *PPM1* knockout was not successful) were included as controls.

**FIGURE 6:**
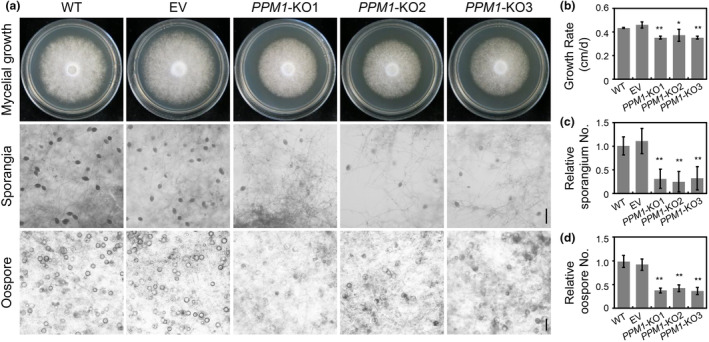
Deletion of *PPM1* significantly affected mycelial growth, sporangia and oospore production of *Phytophthora sojae*. (a) Growth characteristics after 4 days on V8 agar (upper panel: mycelial growth), and microscopic visualization of sporangia (second panel: sporangia) and oospores (third panel: oospore) of the wild‐type (WT), empty vector control line (EV), *PPM1*‐knockout lines (*PPM1*‐KO1, *PPM1*‐KO2 and *PPM1*‐KO3). (b) Growth rates in cm/day on V8 agar. (c) The relative numbers of sporangia and (d) oospores with the number in wild‐type strain (WT) set at 1. Scale bar, 50 μm. Asterisk indicates significant difference, **p < 0.01, *p < 0.05. All experiments were repeated three times with similar results (Table S5).

**FIGURE 7:**
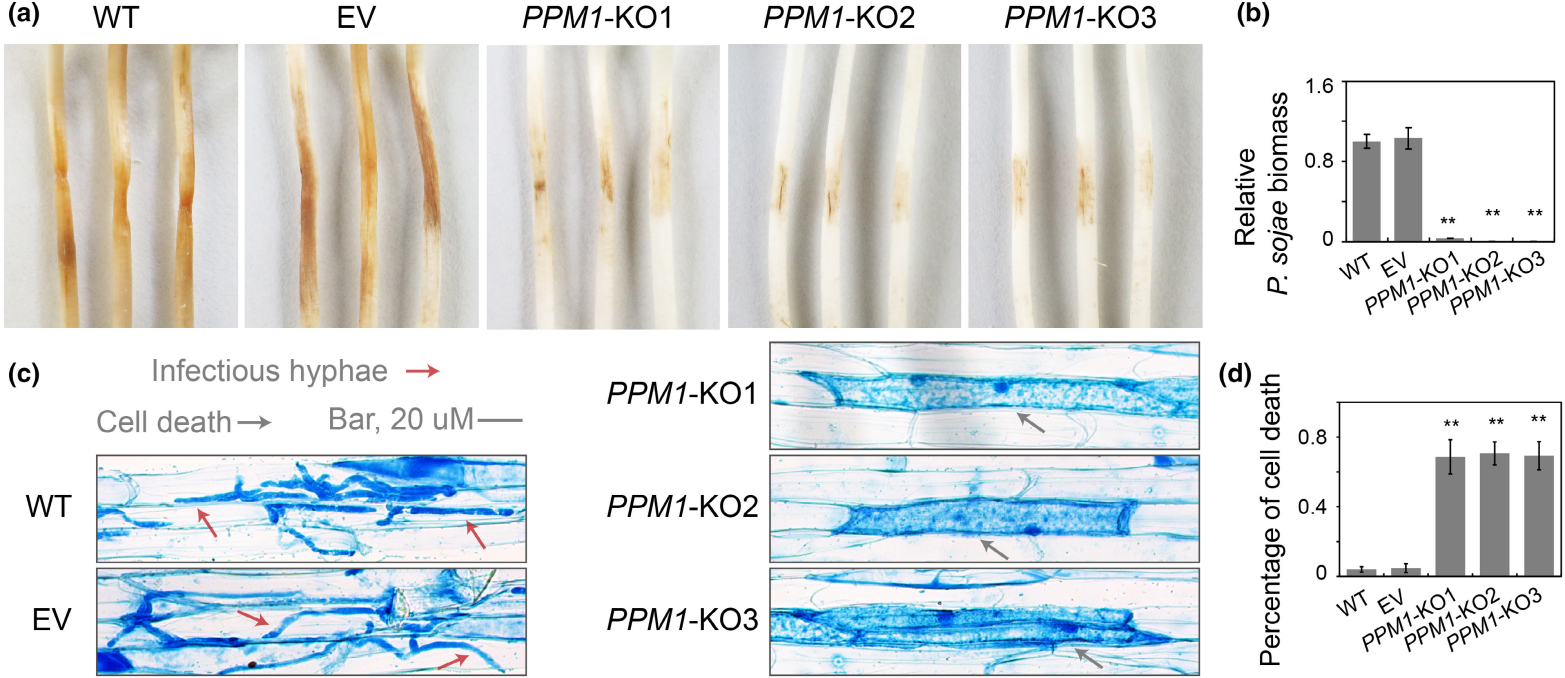
Deletion of *PPM1* significantly affected virulence of *Phytophthora sojae*. (a) Lesions on soybean (cultivar Hefeng 47) at 36 hours post‐inoculation (hpi) of 4‐day‐old etiolated hypocotyls with the wild‐type strain (WT), empty vector control line (EV), *PPM1*‐knockout lines (*PPM1*‐KO1, *PPM1*‐KO2 and *PPM1*‐KO3). (b) Relative pathogen biomass in inoculated etiolated hypocotyls expressed as the ratio between the amounts of *P. sojae* DNA and soybean DNA detected at 36 hpi with the ratio WT:soybean set at 1. (c) Microscopic observations of invasive hyphae in epidermis of soybean hypocotyls at 24 hpi. Trypan blue staining was performed on the epidermis of seedling hypocotyls. Red arrowheads, cysts; grey arrowheads, cell death. Scale bar, 20 μm. (d) Percentage of cell death in epidermal cells of soybean hypocotyls at 24 hpi. In each sample, 50 invasive hyphae were examined. Asterisk indicates significant difference, **p < 0.01, *p < 0.05. All experiments were repeated three times with similar results (Table S5).

Significant differences in growth characteristics were observed when *PPM1* knockout mutants were cultured on V8 medium (Figure 6a,b). With respect to sporangium formation, the WT and EV formed abundant sporangia, while *PPM1*‐KO1, *PPM1*‐KO2 and *PPM1*‐KO3 exhibited a significant reduction (approximately 70%) in number of sporangia (Figure 6a,c). Moreover, an obvious reduction in oospore production was observed in *PPM1* knockout mutants (Figure 6a,d). In addition to these deficiencies in asexual developmental stages, *PPM1* knockout mutants also exhibited obvious impairment in pathogen virulence (Figure 7a,b). Soybean seedlings inoculated with *PPM1* knockout mutants developed only small necrotic lesions at the site of inoculation, whereas WT and EV produced normal disease lesions (Figure 7a). Measurements of relative *P. sojae* biomass in infected soybean seedlings revealed hardly any pathogen biomass in *PPM1* knockout mutants‐infected tissues with levels less than 10% of the levels found in WT‐ or EV‐infected tissues (Figure 7b). In order to investigate the factors underlying the impaired pathogenicity of the *PPM1* knockout strains, epidermal cells from the seedling inoculation sites were excised and subjected to microscopic examination. As depicted in Figure 7c, both the WT and EV strains exhibited a profusion of infectious hyphae that had penetrated into neighbouring cells. In contrast, approximately 70% of epidermal cells within soybean tissue infected by *PPM1*‐KO1, *PPM1*‐KO2 and *PPM1*‐KO3 displayed signs of cell death, as illustrated in Figure 7d. This phenomenon is probably contributing to the decreased frequency of successful *P. sojae* infections.

Taken together, these results demonstrate that the phosphatase PPM1, which contains an N‐terminal PH domain as an accessory domain, was found to be necessary for mycelial growth, sporangia and oospore production and virulence of *P. sojae*. These functional roles of PPM1 are in line with its expression pattern, indicating its essentiality in *P. sojae*. The results of this study demonstrate that PPM1 is an indispensable phosphatase in *P. sojae*.

## 3 DISCUSSION

Many eukaryotic kinases share a common protein kinase‐like fold with proteins found in bacteria, archaea and viruses, which were probably the ancestors of protein kinases. Consequently, kinases can be positioned on an evolutionary tree based on their sequence similarities within species and across phyla (Manning et al., 2002; Miller et al., 2008). In contrast to kinases, phosphatases have distinct evolutionary origins (Andersen et al., 2001; Shi, 2009) and can be categorized into different families. Among these phosphatase families, the conserved CX_5_R motif defines a group that encompasses multiple families (Figure S1). Examples of protein phosphatases within this group include the classical PTPs (PF00102), which are responsible for the dephosphorylation of tyrosine residues, and the CDC25 phosphatase (PF00581), which dephosphorylates tyrosine in cyclin‐dependent kinases (CDK) (Boutros et al., 2007). Additionally, dual specificity phosphatases (PF00782, DSP) dephosphorylate serine, threonine and tyrosine residues (Patterson et al., 2009), while the low molecular weight protein tyrosine phosphatase (PF01451, LMWPTP) (Hoekstra et al., 2015) and SSU72 (PF04722) target serine residues in the C‐terminal domain of RNA polymerase II (Liu et al., 2021). The Cys‐based group includes nonprotein phosphatases, such as Paladin (PF14566), Sac (PF02383) and Myotubularin (PF06602) (Figure S1), which act on lipid substrates (Courtney & Deiters, 2019). Numerous tyrosine‐specific classical PTPs have been identified in animals, whereas in oomycetes and fungi, only a few classical PTPs have been found: three classical PTPs in *P. sojae* and *P. infestans*, and just one in *H. arabidopsidis* (Figure 2a). These findings support the notion that tyrosine kinases are widespread in animals (Pincus et al., 2008), but only two tyrosine kinases have been identified in *P. sojae* and *P. infestans*, with no orthologues found in *H. arabidopsidis* (Judelson & Ah‐Fong, 2010).

In our study, we observed that oomycete species have a significantly higher number of phosphatases in the PPP, PPM and DSP families compared with fungi (Figure 2), all of which are related to serine/threonine phosphatase activity. This finding is particularly significant, and it aligns with previous research in oomycetes that has underscored the importance of serine/threonine phosphatase activity (Qiu et al., 2023). When comparing these two studies, it becomes evident that we have demonstrated the importance of serine/threonine phosphatase activity through two different strategies. In the previous study, we identified a cluster of serine/threonine phosphatase genes based on their specific expression modules among five asexual developmental stages. In the current study, we conducted a comprehensive analysis of all phosphatase genes in oomycetes and fungi, revealing the notably expanded serine/threonine phosphatase activity in oomycetes. These findings collectively contribute to a deeper understanding of the role of serine/threonine phosphatases in oomycetes and further highlight their significance in oomycete biology, which suggests that oomycetes have an increased requirement for dephosphorylation of serine/threonine residues. Supporting this hypothesis, oomycete genomes also contain a large number of tyrosine kinase‐like kinases (TKLs), which have sequence similarity to tyrosine kinases but function as serine/threonine kinases. Oomycetes have hundreds of TKLs, while fungi lack these kinases (Shen et al., 2019). Comparing the kinomes and phosphatomes across different phyla reveals interesting variations. For example, the *Arabidopsis* protein kinome comprises 1125 kinases and 150 phosphatases, with the number of kinases being seven times higher than phosphatases (Tran et al., 2012). In humans, 518 kinases and 189 phosphatases have been reported, with the number of kinases being 2.7 times higher than phosphatases (Chen et al., 2017; Roskoski, 2015). In oomycetes, *P. infestans* has 354 kinases (Judelson & Ah‐Fong, 2010) and 184 phosphatases (Table S1). Here, the number of kinases is 1.92 times higher than phosphatases, indicating a relatively higher ratio of phosphatases in *P. infestans*, primarily due to the expansion of Ser/Thr‐based phosphatases.

In many cases, proteins with accessory domains have been identified in oomycetes, for example kinases (Judelson & Ah‐Fong, 2010) and G‐protein‐coupled receptors (Van den Hoogen et al., 2018). Several studies have confirmed that these proteins with accessory domains involved in signalling or essential biological roles (Sheng et al., 2021; Yang et al., 2013). The findings of this study suggest that a phosphatase with an N‐terminal accessory PH domain plays a critical role in mycelial growth, sporangia and oospore production and virulence in the model oomycete species, *P. sojae*. This PH domain is also present in the human PHLPP1 and PHLPP2 phosphatases, which are involved in apoptosis and the suppression of tumour growth (Brognard et al., 2007). These human PHLPP proteins share a domain composition that includes an N‐terminal PH domain, an LRR region, a phosphatase domain belonging to the PPM family, and a C‐terminal PDZ‐binding motif (Cha et al., 2021). The yeast homologue of PHLPP proteins, Cyr1, lacks the PH and PDZ domains but is fused to adenylate cyclase, while the *Drosophila* homologue retains the LRR, PPM and PDZ ligand but lacks the PH domain (Baker & Kelly, 2004). The PH domain was probably added late in evolution and is present in all mammalian PHLPP isozymes, which includes 90% of living vertebrates (Newton & Trotman, 2014). In oomycetes, the PHLPP homologue retains the PH and PPM domains but lacks the LRR and PDZ domains. In *P. sojae*, this protein is essential for mycelial growth, sporangia and oospore production and virulence (Figure 7). Furthermore, the existence of the PH domain does not affect the phosphatase activity (Figure 6), but its role in substrate specificity and phosphatase activity remains unclear.

This study represents the first extensive catalogue of phosphatases in oomycetes and highlights a crucial phosphatase within the broad serine/threonine phosphatase group in oomycetes. Through detailed analysis, we confirmed that a serine/threonine phosphatase with an accessory domain is indeed an active protein phosphatase. This protein plays indispensable roles in various aspects, including mycelial growth, sporangia and oospore production and infectious stages. Our findings call for more in‐depth functional studies on these phosphatases in pathogenic oomycetes, which significantly contribute to an improved understanding of the functions of phosphatases in oomycetes and may hold promise for the development of innovative strategies to combat oomycete diseases.

## 4 EXPERIMENTAL PROCEDURES

### 4.1 Identification of all phosphatase from oomycete genome

All the proteome data of 22 oomycete and 18 fungal species were obtained from the NCBI database (https://www.ncbi.nlm.nih.gov/). The oomycete species were *Phytophthora ramorum*, *Phytophthora sojae*, *Phytophthora capsici*, *Phytophthora nicotianae*, *Phytophthora parasitica*, *Phytophthora infestans*, *Peronophythora litchii*, *Hyaloperonospora arabidopsidis*, *Peronospora effusa*, *Plasmopara halstedii*, *Pythium vexans*, *Pythium aphanidermatum*, *Pythium arrhenomanes*, *Pythium insidiosum*, *Pythium irregulare*, *Pythium ultimum*, *Albugo candida*, *Albugo laibachii*, *Saprolegnia parastica*, *Achlya hypogyna*, *Aphanomyces astaci* and *Aphanomyces invadans*. Fungi species were *Debaryomyces hansenii*, *Eremothecium gossypii*, *Kluyveromyces lactis*, *Yarrowia lipolytica*, *Saccharomyces cerevisiae*, *Aspergillus terreus*, *Coccidioides immitis*, *Sclerotinia sclerotiorum*, *Fusarium graminearum*, *Fusarium pseudograminearum*, *Fusarium solani*, *Neurospora crassa*, *Fusarium tricinctum*, *Magnaporthe oryzae*, *Verticillium dahlia*, *Cryptococcus neoformans*, *Laccaria bicolor* and *Phycomyces blakesleeanus*.

To identify candidate phosphatase in these species, catalytic sequences from known phosphatase PFAM domains were used to search the proteomes of oomycetes and fungi based on the HMMER program and Hidden Markov Model (HMM). This step allowed us to identify proteins harbouring highly conserved domains in oomycetes or fungi. Next, these newly discovered catalytic sequences were applied to re‐search the proteomes, seeking potential divergent copies of these protein domains. Finally, the found protein sequences were further validated with PFAM database (https://pfam.xfam.org). Domains consistent with the known protein phosphatase family will be retained, and protein family classification were defined according to their different catalytic domains.

### 4.2 Analysis of evolutionary relationship of phosphatase in oomycetes

The whole proteomes data from 22 oomycete and 18 fungal species were applied to obtain single‐copy genes by OrthoFinder (Emms & Kelly, 2019). Then the species phylogeny was constructed through a maximum‐likelihood approach with 1000 bootstrap replicates. Next the phylogenetic trees of all the PPMs from the 22 oomycete and 18 fungal species were constructed using MAFFT for multiple sequence alignment software (Katoh & Standley, 2013), then trimAl for automated alignment trimming (Capella‐Gutierrez et al., 2009), finally IQ‐TREE for phylogenetic inference using maximum likelihood (Minh et al., 2020). Then the gene duplications and losses were analysed by NOTUNG (Chen et al., 2000).

The significant features of the alignment of catalytic domains were analysed by the WebLogo (Crooks et al., 2004).

### 4.3 Analysis of expression patterns and domain combinations of phosphatases

Transcription levels of phosphatase genes were obtained from published RNA‐seq data (Ye et al., 2011). The clustered expression patterns of PPMs were analysed by ClusterGVis (https://github.com/junjunlab/ClusterGVis). The types and positions of domains in PPMs were predicted using CD‐Search (https://www.ncbi.nlm.nih.gov/Structure/bwrpsb/bwrpsb.cgi) and graphically presented using Tbtools (https://github.com/CJ‐Chen/TBtools).

### 4.4 Absorbance‐based detection of serine/threonine phosphatase activity

The method to calculate the activity of Ser/Thr phosphatase was based on the amount of free phosphate released by phosphatase proteins. The free phosphates can be combined by molybdate and malachite green and generate a molybdophosphoric acid complex. The absorbance of this complex at 630 nm was detected to measure the amount of free phosphate in the reaction system.

To obtain purified protein of PPM1, the coding sequences of *PPM1* were inserted into a pET32a vector (containing the His tag). The plasmids (pET32a empty vector and pET32a‐*PPM1*) were transformed to *Escherichia coli* BL21 (DE3), protein expression was induced with 0.1 mM IPTG and after 12 h of growth protein lysates were obtained. The soluble total His‐PPM1 or His‐dePH protein and His protein were incubated with 25 μL His magnetic beads for 8 h at 4°C. Then, the beads were washed three times, and the presence of His proteins was detected by western blotting using anti‐His antibody.

Next the kit of Serine/Threonine Phosphatase Assay System (Promega) was used in this study. To establish a standard curve for free phosphate, phosphate standard dilutions were prepared. Subsequently, reaction premixes were prepared in 96‐well plates. Purified protein samples were added to initiate the reactions. After 15 min, an equal volume of the molybdate dye/additive mixture was added to all wells to halt the reactions. Finally, the amount of free phosphates in each protein sample was determined by measuring the absorbance at 630 nm. This allowed for the quantification of free phosphates in the protein samples.

### 4.5 CRISPR/Cas9‐mediated gene knockout

Two sgRNAs (sgRNA1 and sgRNA2, Table S4) were designed for targeting the *PPM1* open reading frame. The precise replacement of the gene was achieved through homology‐directed repair (HDR), wherein the donor DNA comprised the *Hygromycin* open reading frame ligated to two 1 kb fragments flanking the *PPM1* gene. The plasmid vector used for delivering the HDR template was pBluescript SK II^+^ (pBS‐SK II^+^). The polyethylene glycol‐mediated protoplast transformation method previously described was employed for the transformation of *P. sojae* (Hua et al., 2008).

Genomic DNA from potential transformants was extracted, and two rounds of primers were used in genomic PCR to validate the HDR mutants (Figure S4a, Table S4). In the first round, F1/R1 primers (Figure S4a, Table S4), located within the *PPM1* open reading frame, were used to detect deletion events. The absence of *PPM1* bands in the *PPM1* knockout mutants (*PPM1*‐KO1/KO2/KO3) indicated the occurrence of deletion events (Figure S4b, F1/R1). Subsequently, the second round of PCRs employed primers situated outside the *PPM1* homology arms (to avoid interference from transferred plasmids) and within the *Hygromycin* gene to detect homologous recombination events. As depicted in Figure S4b (F2/R2, F2/R3), the *PPM1* knockout mutants exhibited clear bands of replaced sequences, signifying the successful replacement of the original *PPM1* open reading frame in the *P. sojae* genome with the *Hygromycin* open reading frame. Sanger sequencing across the junctions of the flanking sequences and *Hygromycin* gene consistently supported the replacement of the *PPM1* open reading frame with the *Hygromycin* gene (Figure S4a).

### 4.6 Growth, oospore and sporangium production

To determine growth rate, all strains were grown on V8 medium at 25°C in the dark. Colony diameters were measured over 4 days, and the average growth rates were calculated (mm/day).

To monitor and quantify oospore production, strains were grown on lima bean agar (LBA) at 25°C in the dark. After 10 days the cultures were examined by microscopy. Three blocks of 1 cm^2^ were cut from the medium and in each block three random fields at 40× magnification were selected for counting the number of oospores.

To quantify sporangia production 10% V8 broth was inoculated with three mycelial disks (7 mm in diameter) cut from a culture and incubated for 48 h at 25°C in the dark. The mycelia were then rinsed twice with sterile distilled water, then flooded with sterile distilled water and left for 8 h to stimulate sporangia formation. This was then gently mixed in a blender to obtain a homogenous mixture. Subsequently, three random samples of 100 μL were taken and by microscopic examination at 40× magnification the number of sporangia in each sample was counted. All assays were repeated at least three times.

### 4.7 Virulence assays of *P. sojae* strains on soybeans

The soybean cultivar Hefeng 47, which is susceptible to *P. sojae* P6497, was grown in plastic pots containing vermiculite at 25°C for 4 days in the dark.

Zoospores were obtained as described in sporangia production and diluted to 50 zoospores/10 μL. Etiolated seedlings were inoculated by 10 μL of zoospores on the hypocotyls and maintained in a climate‐controlled room at 25°C and 80% relative humidity in the dark. Manifestations of virulence were evaluated at 36 h post‐inoculation (hpi), and photographs were taken. Each strain was tested using at least three different preparations of mycelium pellets and five plants. All assays were repeated at least three times.

To facilitate microscopic observation of the infection situation within soybean tissue, we collected infected epidermal cells at 24 hpi and subjected them to trypan blue staining. Following destaining procedures involving chloral hydrate and water, the infected epidermal cells were meticulously examined using a light microscope. Each strain was tested using at least two different preparations of zoospores and five plants. All assays were repeated at least three times.

## CONFLICT OF INTEREST STATEMENT

The authors declare that they have no conflict of interest.

## Supporting information


**Figure S1.** Catalytic domains of phosphatases defined from different species. The phosphatase families, their Pfam IDs and their reported substrate preference. AP, alkaline phosphatase; CDC, cell division cycle; DSP (or DUSP), dual‐specificity phosphatase; HAD, haloacid dehalogenase; HP, histidine phosphatase; LMPTP, low molecular mass PTP; PPM, metallo‐dependent protein phosphatase; PPP, phosphoprotein phosphatase; PTP, protein tyrosine phosphatase; RTR1, regulator of transcription 1.

**Figure S2.** The distribution of transcript tags across five gene transcripts. These five genes, *Ps354449* (a), *Ps568223* (b), *Ps480075* (c), *Ps544740* (d) and *Ps313333* (e) are predicted to encode phosphatases with accessory kinase domains. The read mapping data supports that each of these genes has a single mRNA transcript.

**Figure S3.** Phosphatase activity of *Phytophthora sojae* PPM1 without PH domain. (a) The purified protein of negative control (His) and His tag‐fused PPM1 without PH domain (His‐dePH). The empty pET32a vector contains Trx tag, S tag and His tag, thus the negative control (His) encodes a 20 kDa protein. The His‐dePH encode a fusion protein about 84 kDa. The presence of His‐tagged proteins was detected by western blot analysis using an anti‐His antibody. (b) The amount of free phosphate that released by the negative control (His), His tag‐fused PPM1 without PH domain (His‐dePH) and His‐dePH mixed with chelating agent (EDTA). The experiments were repeated three times with similar results (Table S5).

**Figure S4.** Screening of knockout mutants of PPM1. (a) Locations of the primers used to screen the knockout mutants (F1/R1, F2/R2, and F3/R3) are indicated. Sanger sequencing traces of junction regions confirming that the *PPM1* open reading frame was precisely replaced. Red dots, junction regions. (b) Analysis of genomic DNA from the wild‐type (WT), control line (EV), and *PPM1*‐knockout lines (*PPM1*‐KO1, *PPM1*‐KO2 and *PPM1*‐KO3) using the primers in (a) and actin primers (positive control).

**Table S1.** Numbers of phosphatases in 22 oomycetes and 18 fungal species.

**Table S2.** All the sequences of phosphatases identified in 22 oomycetes and 18 fungal species.

**Table S3.** Domain annotations of proteins included in Figure 4.

**Table S4.** Primers used in this study.

**Table S5.** The numerical data pertaining to experimental repeats in this study.

## Data Availability

The data that supports the findings of this study are available in the supplementary material of this article.
