# Supplementary figures and images for: Mining oomycete proteomes for phosphatome leads to the identification of specific expanded phosphatases in oomycetes
Source: Mol Plant Pathol. 2024 Mar 10;25(3):e13425. doi: 10.1111/mpp.13425 (PMC10925823; doi:10.1111/mpp.13425)

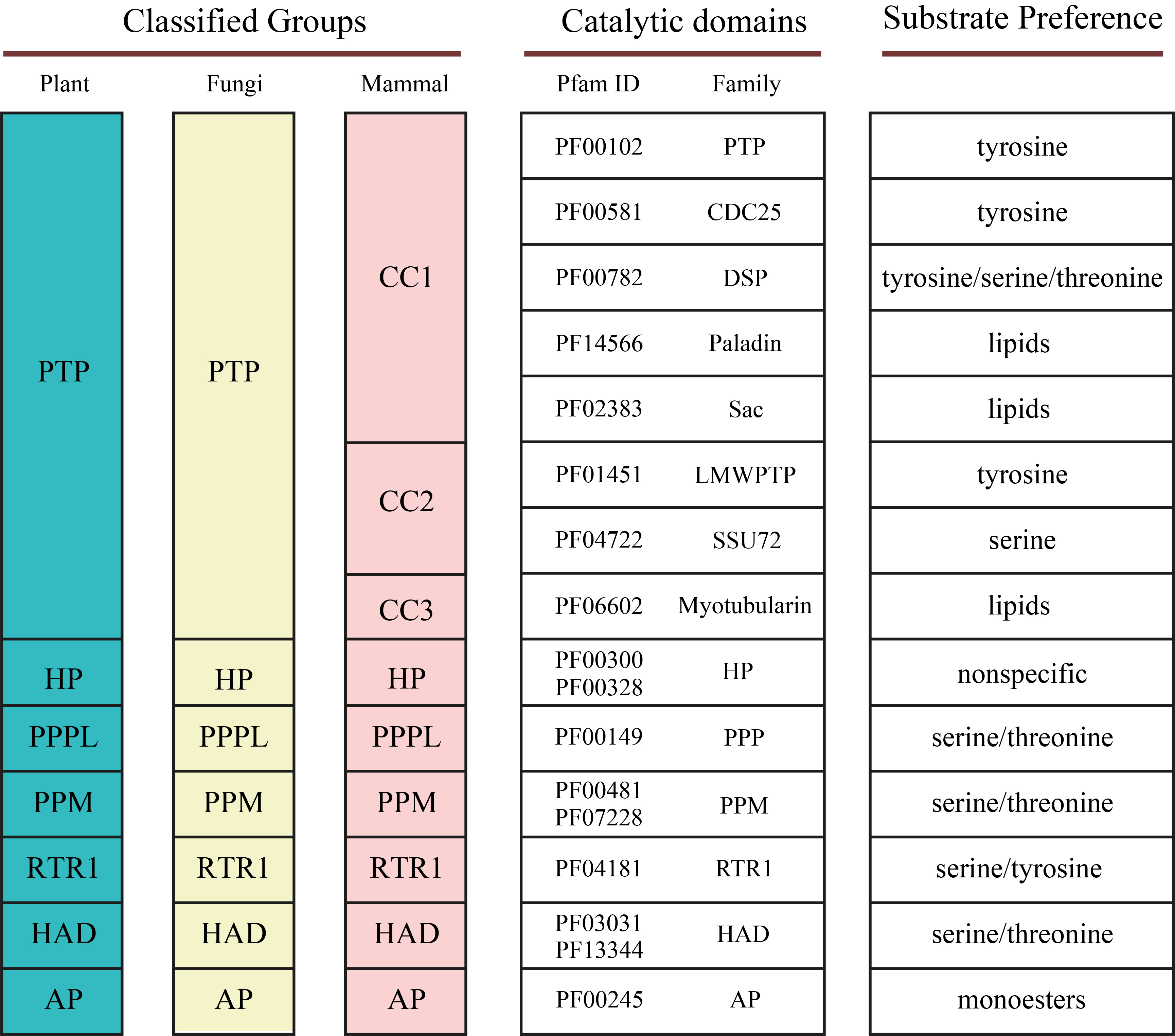

Supplement: Supplementary file 1 — Figure S1. Catalytic domains of phosphatases defined from different species. The phosphatase families, their Pfam IDs and their reported substrate preference. AP, alkaline phosphatase; CDC, cell division cycle; DSP (or DUSP), dual‐specificity phosphatase; HAD, haloacid dehalogenase; HP, histidine phosphatase; LMPTP, low molecular mass PTP; PPM, metallo‐dependent protein phosphatase; PPP, phosphoprotein phosphatase; PTP, protein tyrosine phosphatase; RTR1, regulator of transcription 1. [file MPP-25-e13425-s004.jpg]

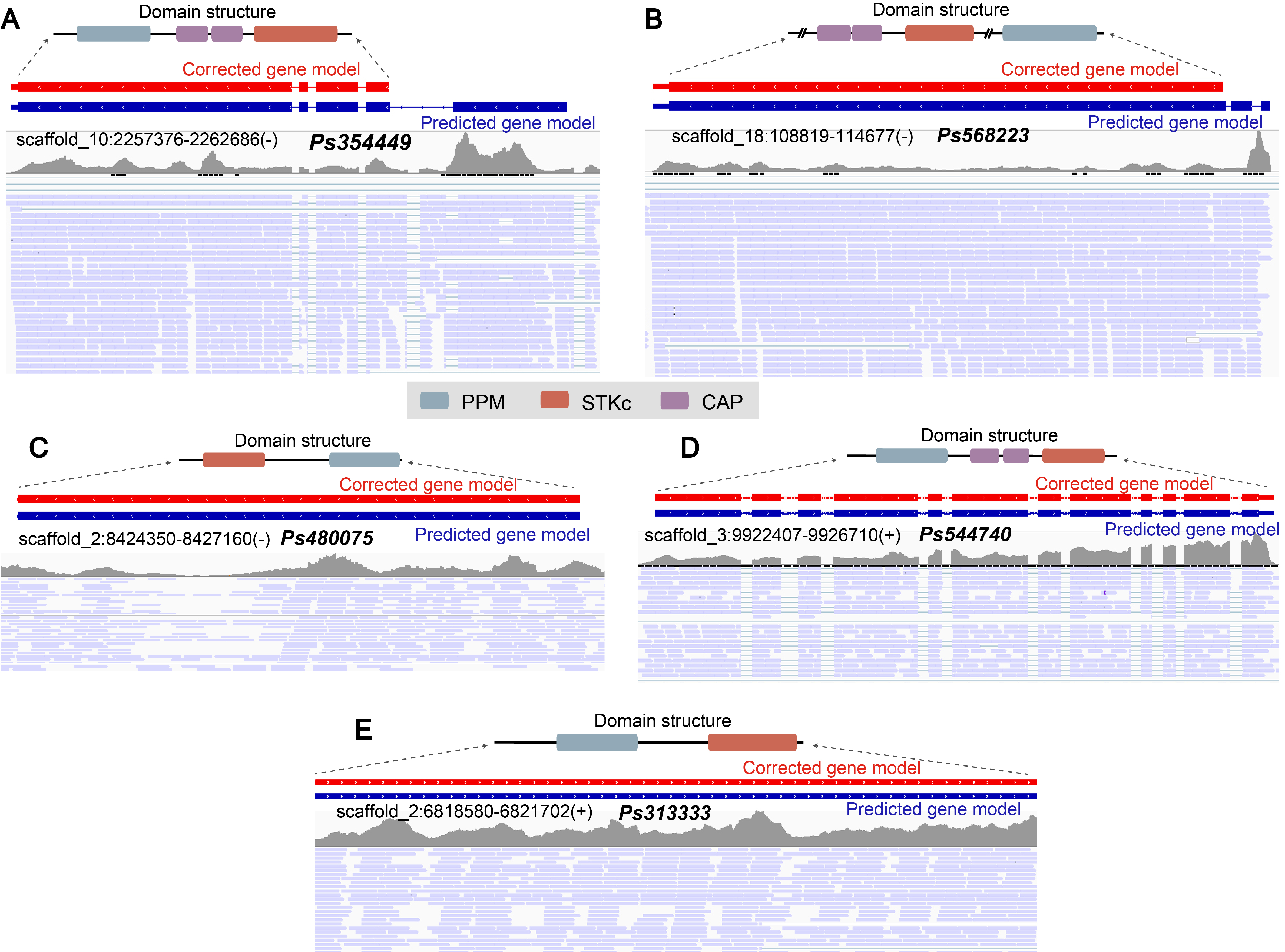

Supplement: Supplementary file 2 — Figure S2. The distribution of transcript tags across five gene transcripts. These five genes, Ps354449 (a), Ps568223 (b), Ps480075 (c), Ps544740 (d) and Ps313333 (e) are predicted to encode phosphatases with accessory kinase domains. The read mapping data supports that each of these genes has a single mRNA transcript. [file MPP-25-e13425-s003.jpg]

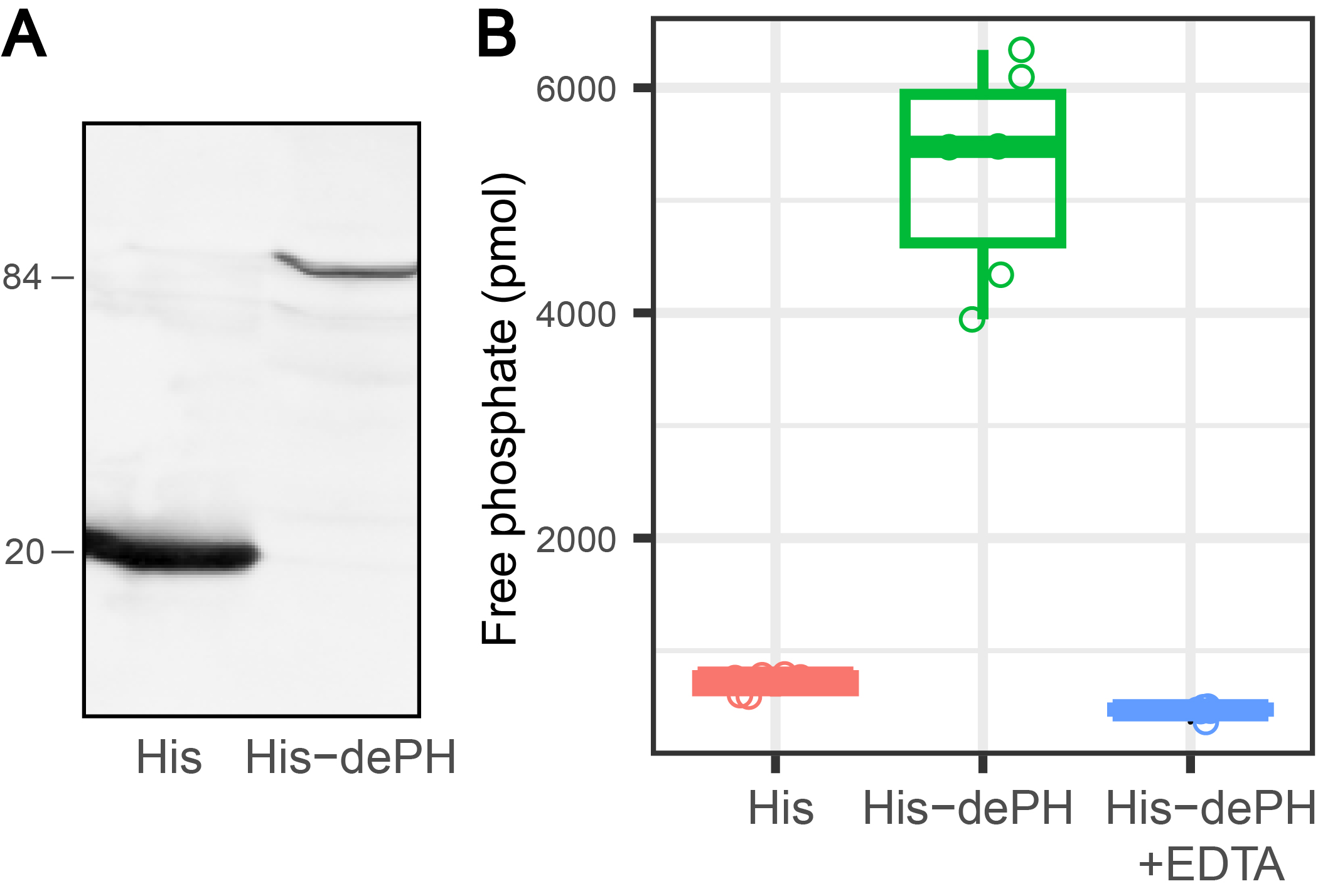

Supplement: Supplementary file 3 — Figure S3. Phosphatase activity of Phytophthora sojae PPM1 without PH domain. (a) The purified protein of negative control (His) and His tag‐fused PPM1 without PH domain (His‐dePH). The empty pET32a vector contains Trx tag, S tag and His tag, thus the negative control (His) encodes a 20 kDa protein. The His‐dePH encode a fusion protein about 84 kDa. The presence of His‐tagged proteins was detected by western blot analysis using an anti‐His antibody. (b) The amount of free phosphate that released by the negative control (His), His tag‐fused PPM1 without PH domain (His‐dePH) and His‐dePH mixed with chelating agent (EDTA). The experiments were repeated three times with similar results (Table S5). [file MPP-25-e13425-s008.jpg]

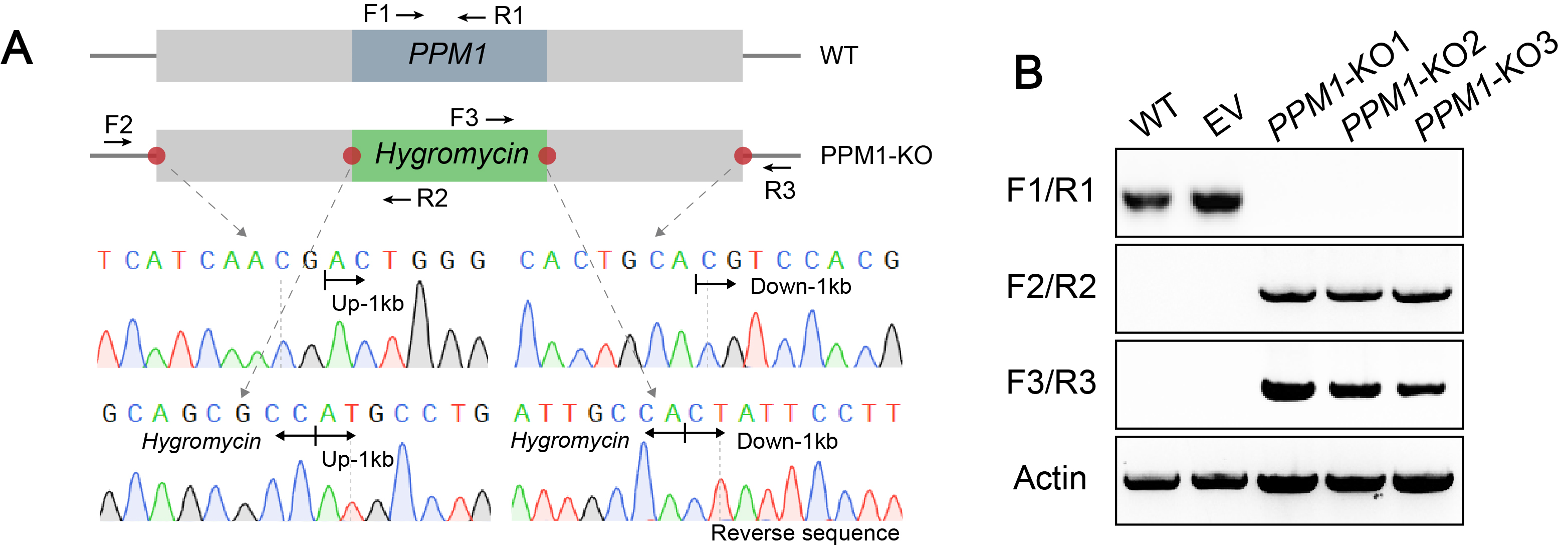

Supplement: Supplementary file 4 — Figure S4. Screening of knockout mutants of PPM1. (a) Locations of the primers used to screen the knockout mutants (F1/R1, F2/R2, and F3/R3) are indicated. Sanger sequencing traces of junction regions confirming that the PPM1 open reading frame was precisely replaced. Red dots, junction regions. (b) Analysis of genomic DNA from the wild‐type (WT), control line (EV), and PPM1‐knockout lines (PPM1‐KO1, PPM1‐KO2 and PPM1‐KO3) using the primers in (a) and actin primers (positive control). [file MPP-25-e13425-s001.jpg]
